# Polytrauma patients with severe cervical spine injuries are different than with severe TBI despite similar AIS scores

**DOI:** 10.1038/s41598-022-25809-8

**Published:** 2022-12-13

**Authors:** Karlijn J. P. van Wessem, Menco J. S. Niemeyer, Luke P. H. Leenen

**Affiliations:** 1 Department of Trauma Surgery, University Medical Center Utrecht, Utrecht, The Netherlands; 2 Department of Trauma Surgery, University Medical Center Utrecht, Suite G04.232, Heidelberglaan 100, 3584 CX Utrecht, The Netherlands

**Keywords:** Neurology, Trauma

## Abstract

Traumatic cervical spine injuries (TCSI) are rare injuries. With increasing age the incidence of TCSI is on the rise. TCSI and traumatic brain injury (TBI) are often associated. Both TCSI and TBI are allocated to the Abbreviated Injury Scale (AIS) head region. However, the nature and outcome of these injuries are potentially different. Therefore, the aim of this study was to investigate the epidemiology, demographics and outcome of severely injured patients with severe TCSI, and compare them with polytrauma patients with severe TBI in the strict sense. Consecutive polytrauma patients aged ≥ 15 years with AIShead ≥ 3 who were admitted to a level-1 trauma center Intensive Care Unit (ICU) from 2013 to 2021 were included. Demographics, treatment, and outcome parameters were analyzed for patients who had AIShead ≥ 3 based on TCSI and compared to patients with AIShead ≥ 3 based on proper TBI. Data on follow-up were collected for TCSI patients. Two hundred eighty-four polytrauma patients (68% male, Injury Severity Score (ISS) 33) with AIShead ≥ 3 were included; Thirty-one patients (11%) had AIShead ≥ 3 based on TCSI whereas 253 (89%) had AIShead ≥ 3 based on TBI. TCSI patients had lower systolic blood pressure in the Emergency Department (ED) and stayed longer in ICU than TBI patients. There was no difference in morbidity and mortality rates. TCSI patients died due to high cervical spine injuries or respiratory insufficiency, whereas TBI patients died primarily due to TBI. TCSI was mainly located at C2, and 58% had associated spinal cord injury. Median follow-up time was 22 months. Twenty-two percent had improvement of the spinal cord injury, and 10% died during follow-up. In this study the incidence of severe TCSI in polytrauma was much lower than TBI. Cause of death in TCSI was different compared to TBI demonstrating that AIShead based on TCSI is a different entity than based on TBI. In order to avoid data misinterpretation injuries to the cervical spine should be distinguished from TBI in morbidity and mortality analysis.

## Introduction

Traumatic cervical spinal injuries (TCSI) are a rare entity in the general trauma population with a historically bimodal distribution with peaks in younger people around their 20s and elderly in their 60–70s. With increasing age of the general trauma population, the incidence of TCSI has increased (with conservation of the bimodal distribution), and an incidence up to 6.7% has been reported in trauma patients^1–4^. Additionally, the incidence of moderate to severe traumatic brain injury (TBI) is on the rise^5^. In a previous study it was demonstrated that the proportion of TBI has become the most common cause of death in blunt trauma^6^. In literature, the incidence of cervical spine injury in patients with TBI has been reported up to 13%^7–10^. Conversely, TBI is frequently associated with cervical spinal injury; the incidence of moderate to severe TBI in patients with cervical spine injury is reported between 18 and 40%^4,7^. Additionally, recent studies estimated that concurrent TBI in patients with primary traumatic spinal cord injury ranged from 12.5 to 74.2%, based on the diagnostic criteria utilized^11,12^.

Injury severity score (ISS) is an anatomical scoring system that provides an overall score for patients with multiple injuries. Each injury is assigned an abbreviated injury scale (AIS) score and is allocated to one of six body regions. ISS is calculated by taking the highest AIS severity code in each of the three most severely injured ISS body regions, square each AIS code and add the three squared numbers for an ISS. When calculating data on ISS in trauma patients, TBI and injury to the cervical spine are both part of AIShead, since AIS scores are created to assess the injury severity including the threat to life associated with the injury based on anatomical location^13^. However, the nature and outcome of these injuries are potentially different. Therefore, the aim of this study was to investigate the epidemiology, demographics and outcome of severely injured patients with severe TCSI (AIShead ≥ 3), and compare them with polytrauma patients with severe TBI in the strict sense. It was hypothesized that there was no difference in morbidity and (cause of) mortality between polytrauma patients with associated severe TCSI and severe TBI.

## Methods

### Study setting

From November 2013–November 2021 a prospective population-based cohort study was undertaken including all consecutive polytrauma patients with AIShead ≥ 3 who were admitted to the Intensive Care Unit (ICU) of the University Medical Center Utrecht. This is a level-1 trauma center in the province of Utrecht and covers the central region of the Netherlands with a service area of 1500 square kilometers and approximately 1.3 million residents. The service area for neurosurgery facilitates 2.1 million residents. Around 1300 trauma patients with full activation of a trauma team are annually admitted. Approximately 375 of them are polytrauma patients^14^. Polytrauma (severely injured) patients included in the study were all admitted to ICU either directly from the emergency department (ED) or postoperatively after urgent surgery was performed. Patients < 16 years of age, and/or with isolated injury to the brain (AIShead 3 or more and AIS 2 or less in other regions), asphyxiation, hanging, drowning and burns were excluded, because of possible different physiologic response to severe trauma and a significantly different mortality and morbidity profile^15,16^. Patients who had both severe TCSI and severe TBI (AIShead ≥ 3) were also excluded from further analysis.

A polytrauma patient was defined as a patient with AIS score of greater than 2 in at least two ISS body regions combined with the presence of one or more physiological risk factors such as hypotension (systolic blood pressure ≤ 90 mmHg), level of consciousness (Glasgow Coma Scale score ≤ 8), acidosis (base excess ≤  − 6.0 mEq/L), coagulopathy (international normalized ratio ≥ 1.4/partial thromboplastin time ≥ 40 s) and age (≥ 70 years)^17^.

### Data collection

All in-hospital data were prospectively collected on arrival in ED and on a daily basis in ICU by authors KW and LL, and included patient demographics, ISS, shock and resuscitation parameters. Both crystalloid and blood product (Packed Red Blood Cells (PRBC), Fresh Frozen Plasma (FFP), Platelets (PLT)), and tranexamic acid (TXA) administration was recorded in the first 24 h following admission. Denver Multiple Organ Failure (MOF) scores and Adult Respiratory Distress Syndrome (ARDS) Berlin criteria were registered daily up until 28 days or discharge from ICU^18,19^. All severely injured patients had a total body CT scan on arrival in ED. Cervical spinal injuries were classified based on the level of the bony injury. In case of suspicion of ligamentous spinal injury and/or spinal cord injury, an additional magnetic resonance imaging (MRI) was performed to evaluate the spinous ligaments and spinal cord. Spinal cord injury was quantified by the American Spinal Injury Association (ASIA) impairment scale^20^.

There were two causes of death in which patients developed failure of the respiratory system; Patients with high cervical spinal cord injuries (above C5) and the inability to breath spontaneously who subsequently died of respiratory failure were classified as death by high cervical spinal injury. In contrast, to avoid confusion between the two causes of death, patients who died because of respiratory failure caused by injuries to the chest and/or pulmonary complications such as pneumonia were classified as death by respiratory failure.

Follow-up data in polytrauma patients with associated cervical spine injury were retrospectively collected and follow-up time was calculated up until 31st January 2022.

Primary outcome was the comparison of polytrauma patients with associated AIShead ≥ 3 based on TCSI and TBI. Additionally, detailed data on the epidemiology and outcome of TCSI patients was collected.

### Ethical approval

The Medical Ethical Review Board of the University Medical Center Utrecht approved this study and granted a waiver of informed consent (reference number WAG/mb/16/026664). All methods were performed in accordance with the relevant guidelines and regulations.

### Statistical analysis

Data were analyzed using IBM SPSS Statistics, version 25.0 (Armonk, NY, USA). Results are presented as median and interquartile range (IQR). Comparison of continuous variables was done using Kruskal–Wallis. Significant differences for categorical variables were calculated through Chi-Square test or Fisher’s exact test depending on the size of the groups. Statistical significance was defined as P < 0.05.

## Results

**Figure 1:**
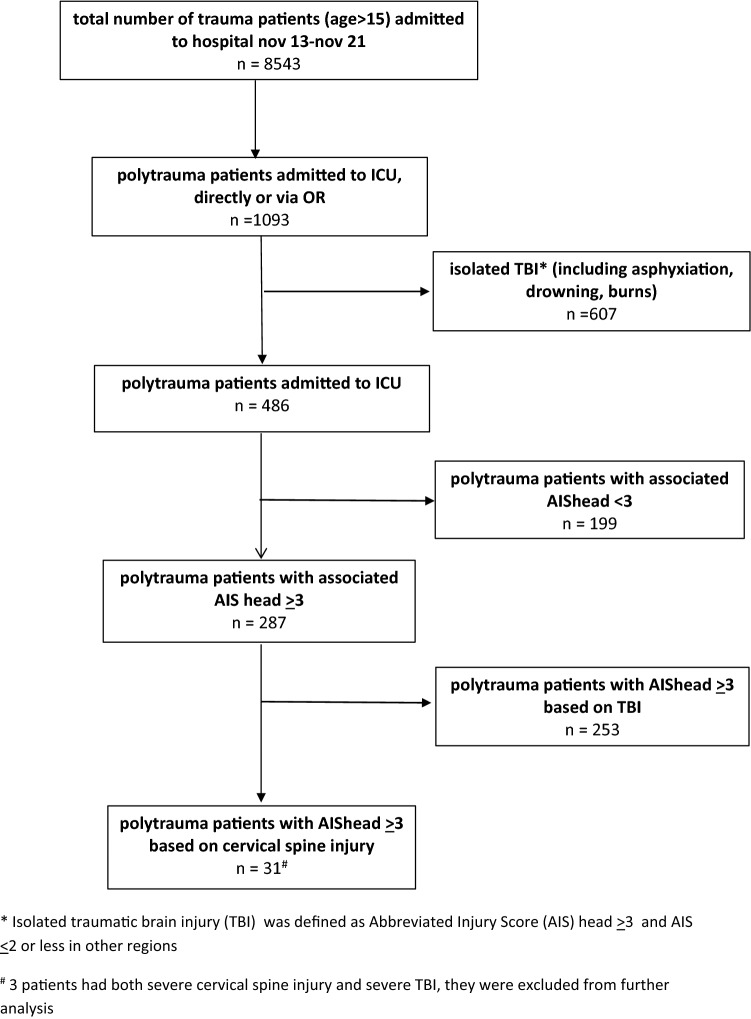
Flowchart of included patients.

Two hundred eighty-seven severely injured patients (68% male, ISS 33) with AIShead ≥ 3 who were admitted to ICU were included. A flowchart of included patients is shown in Fig. 1. Two hundred fifty-three patients had AIShead ≥ 3 based on TBI in the strict sense, whereas 31 patients had AIShead ≥ 3 based on TCSI. Three patients who had both severe TCSI and severe TBI (AIShead ≥ 3) were excluded, so two hundred eighty-four patients were included for further analysis. Physiology, resuscitation, and outcome data of both patient groups are presented in Table 1. Patients who had AIShead ≥ 3 based on TCSI had a lower AIShead (3 (3–5) vs. 4 (3–4), P < 0.001), and lower AISpelvis/extremities (0 (0–3) vs. 2 (1–3), P = 0.008). Both groups had associated severe chest injuries; Seventy-one % (22/31) of TCSI patients and 72% (183/253) had a serious associated thoracic injuries (AISchest ≥ 3).

**Table 1:** Demographics in polytrauma with associated AIS head ≥ 3.

	AIS head ≥ 3 based on cervical spine injury (n = 31)	AIS head ≥ 3 based on TBI (n = 253)	P-value
Age (years)	55 (32–73)	48 (28–63)	0.10
Male gender	22 (71%)	172 (68%)	0.74
Blunt MOI	30 (97%)	247 (98%)	0.56
Prehospital intubation	16 (52%)	153 (62%)	0.26
ISS	29 (22–50)	33 (27–38)	0.94
AIS head	3 (3–5)	4 (3–4)	< 0.001*
AIS face	0 (0–1)	0 (0–2)	0.54
AIS chest	3 (2–4)	3 (2–4)	0.88
AIS abdomen	0 (0–2)	0 (0–2)	0.08
AIS pelvis/extremities	0 (0–2)	2 (1–3)	0.008*
AIS external	0 (0–1)	0 (0–1)	0.40
SBP_ED (mmHg)	100 (80–124)	124 (101–142)	< 0.001*
SBP ≤ 90 mmHg_ED	14 (45%)	41 (16%)	< 0.001*
Hb_ED (mmol/L)	7.8 (7.1–8.3)	8.0 (7.2–8.9)	0.22
pH_ED	7.31 (7.22–7.38)	7.31 (7.24–7.36)	0.85
PaCO_2__ED (mmHg)	46 (38–56)	46 (42–53)	0.71
BD_ED (mEq/L)	− 4.0 (− 7.0 to 0.0)	− 3.0 (− 6.0 to 0.0)	0.97
PT_ED (s)	14.7 (13.4–15.5)	14.2 (12.7–16.2)	0.60
Temperature_ED (°C)	36.1 (35.2–36.7)	35.5 (34.6–36.5)	0.56
SBP_ICU (mmHg)	122 105–134)	119 (108–135)	0.75
Hb_ICU (mmol/L)	7.6 (6.8–8.2)	7.6 (6.8–8.3)	0.66
pH_ICU	7.36 (7.28–7.39)	7.33 (7.28–7.38)	0.40
PaCO_2__ICU (mmHg)	40 (36–46)	42 (38–46)	0.26
BD_ICU (mmol/L)	− 4.8 (− 6.9 to − 1.7)	− 3.8 (− 6.3 to − 2.0)	0.52
Temperature_ICU (°C)	34.9 (33.8–35.9)	35.3 (34.3–36.0)	0.23
UO_ICU (mL)	210 (80–413)	165 (100–376)	0.94
**Resuscitation parameters**			
Crystalloids ≤ 24 h (L)	6.5 (5.7–8.1)	7.1 (4.6–9.8)	0.71
PRBC ≤ 24 h (U)	1 (0–4)	0 (0–4)	0.90
PRBC ≥ 10 units ≤ 24 h	2 (7)	21 (8)	1.0
FFP ≤ 24 h (U)	0 (0–4)	0 (0–4)	0.66
PLT ≤ 24 h (U)^#^	0 (0–0)	0 (0–1)	0.70
TXA	19 (61%)	159 (63%)	0.87
**Outcome**			
Ventilator days	7 (3–17)	6 (3–11)	0.26
Ventilator free days	10 (1–18)	12 (1–19)	0.77
ICU LOS (days)	13 (5–23)	7 (3–14)	0.003*
H-LOS (days)	23 (15–37)	20 (9–33)	0.14
MODS	7 (23%)	38 (15%)	0.28
ARDS	1 (3%)	5 (2%)	0.50
Infectious complications	16 (52%)	105 (42%)	0.34
Thrombo-embolic complications	2 (7%)	14 (6%)	0.69
In-hospital mortality	5 (16%)	61 (24%)	0.38
GOS	3 (3–3)	3 (2–3)	0.45

Data are expressed as median (IQR) or absolute numbers (%).

*TBI* traumatic brain injury, *MOI* mechanism of injury, *ISS* injury severity score, *AIS* Abbreviated Injury Scale, *ED* Emergency Department, *SBP* systolic blood pressure, *Hb* hemoglobin, *PaC02* partial pressure of carbon dioxide in arterial blood, *BD* base deficit, *PT* prothrombin time, *UO* urinary output first hour in ICU, *PRBC* packed red blood cells, *FFP* fresh frozen plasma, *PLT* platelets, *TXA* tranexamic acid, *LOS* length of stay, *MODS* multiple organ dysfunction syndrome, *ARDS* adult respiratory distress syndrome, *GOS* Glasgow Outcome Score.

*statistically significant.

**Figure 2:**
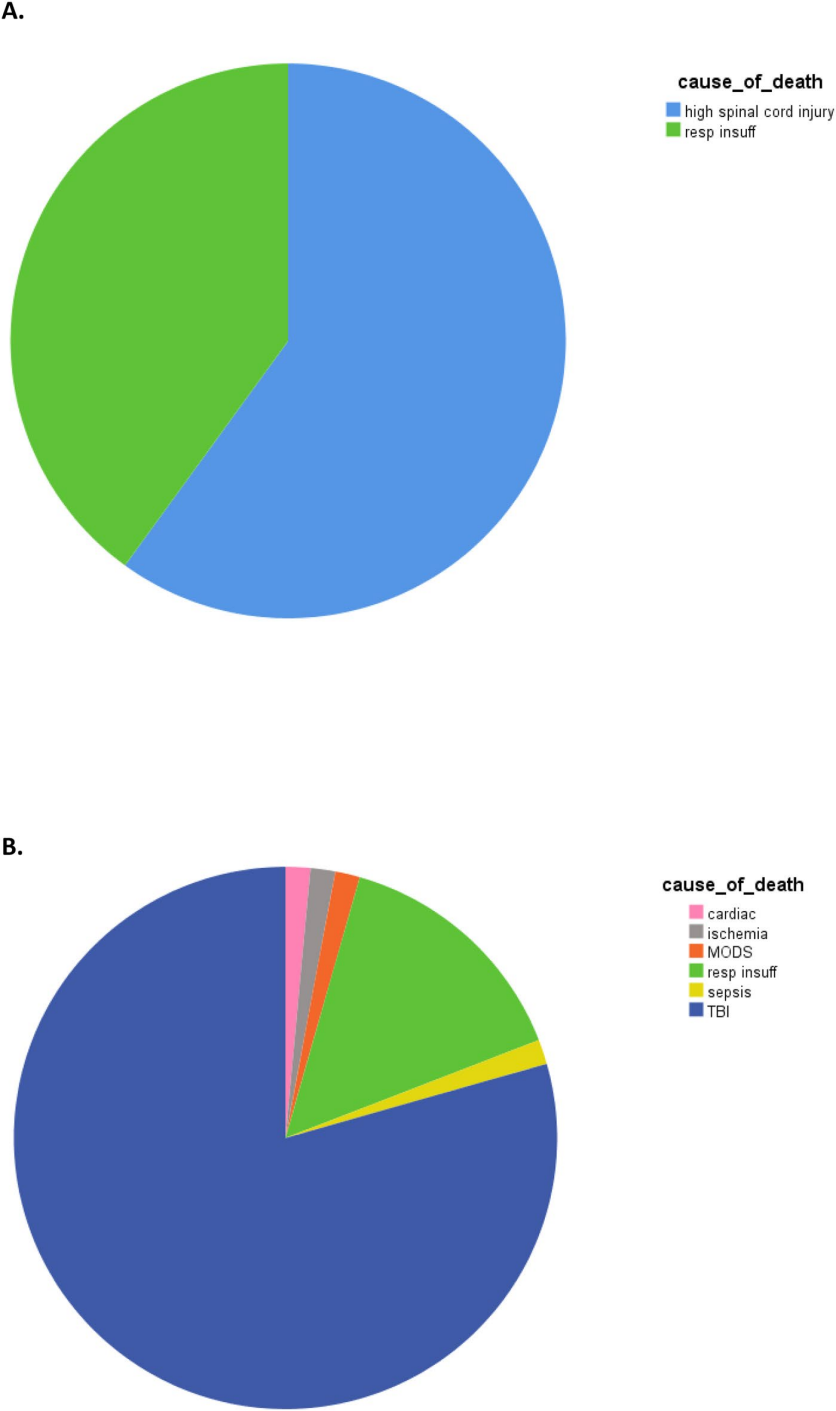
Cause of in-hospital death in polytrauma patients with (**A**) associated severe cervical spine injury, or (**B**) with associated severe traumatic brain injury.

Further, TCSI patients had a lower systolic blood pressure (SBP) on arrival in ED (SBP_ED 100 (80–124) vs. 124 (101–142) mmHg, P < 0.0001). There were no differences in other physiological parameters. There was also no difference in the administration of crystalloids nor in blood products in the first 24 h between both groups. Patients with associated severe TCSI stayed longer in ICU, but had similar days on the ventilator and length of hospital stay. There was no difference in complication rate and mortality (Table 1). Median time of death in TBI was 6 (2–11) days after admission compared to 11 (2–47) days in TCSI patients (P = 0.18). There was however a difference in cause of death between both groups; patients with associated TCSI died mainly of inability to breath spontaneously caused by the spinal cord injury on the level on C2–C3, followed by respiratory insufficiency (due to injuries to the chest or pulmonary complications, but not primarily caused by spinal cord injury), whereas the vast majority of patients with associated TBI died of the brain injury, followed by respiratory insufficiency (Fig. 2).

### Traumatic cervical spine injury location

The most common cervical spine injury was located in C2 (n = 16, 29%), followed by C6 and C7 (n = 11, (18%) and n = 12, (22%), respectively). Three patients (5%) had injuries located in C1, four patients (7%) in C3, six patients (11%) in C4, and five patients (9%) in C5. Nineteen (61%) patients with severe TCSI had injuries on multiple levels in the cervical spine. Twenty patients (65%) who had cervical spine injuries also had injuries in thoracic (16 patients) and/or lumbar spine (5 patients, one of them had injuries in both thoracic and lumbar spine, Table 2). Two TCSI patients (6%) had associated minor TBI (AIShead ≤ 2).

**Table 2:** Characteristics of cervical spine specific injury.

Cervical spine characteristics	N = 31
Multiple level cervical spine injury	20 (65)
**Associated spinal cord injury**	18 (58)
ASIA A	10
ASIA B	2
ASIA C	3
ASIA D	3
ASIA E	13
Surgical fixation of cervical spine	15 (48)
Associated thoracic spine injuries	16 (52)
Associated lumbar spine injuries	5 (16)*
Associated minor TBI (AIShead ≤ 2)	2 (6)

Data are expressed in absolute numbers (%).

*ASIA* American Spinal Injury Association, *TBI* traumatic brain injury, *AIS* Abbreviated Injury Scale.

*1 patient had injuries in both thoracic and lumbar spine.

Five patients suffered from associated blunt cerebrovascular injuries to either internal carotid arteries or vertebral arteries. They were treated with antiplatelet therapy without further complications.

Twenty-one (68%) patients underwent urgent surgery (≤ 24 h), thirteen of them had spinal fixation within 24 h. Another two patients had spinal fixation on respectively day 1 and day 3 after admission. Eight patients had urgent surgery for other injuries (abdominal injuries, extremity fractures, Table 3).

**Table 3:** Type and timing of surgery.

Surgical procedure^#^	Urgent surgery ≤ 24 h N = 21	Surgery > 24 h N = 21	Total
Laparotomy	6	0	6
Abdominal closure	0	4	4
Spine fixation	13	2	15
Ex fix pelvis/extremity	4	0	4
Fracture fixation	2	9	11
Tracheotomy	1	4	5
Miscellaneous*	1	1	2
Total	27	20	47

^#^Several patients underwent more than 1 surgical procedure during 1 session in the operating room.

*Miscellaneous surgeries included eye exploration, soft tissue debridement.

### Traumatic spinal cord injury (TSCI)

Eighteen patients (65%) had associated traumatic spinal cord injury (TSCI), and more than half of patients sustained a complete spinal cord injury (ASIA A, Table 2). C3–C4 was the most frequent location of TSCI (Table 4). All patients with C1 and C2 fractures with associated TSCI died in hospital because of high cervical spinal cord injury and the inability to breath spontaneously. Twelve TSCI patients (67%) had fixation of their spinal fractures, all within 24 h after arrival in ED. Six patients had no spinal fixation; three of them died early after admission due to high cervical spinal cord injury, one other patient had a penetrating injury to C5, and two others had minimal osseous injuries that needed no surgical stabilization. There was no difference in physiological and resuscitation parameters nor any difference in morbidity and in-hospital mortality compared to patients without TSCI.

**Table 4:** Location and severity of traumatic spinal cord injury (TSCI).

	ASIA A	ASIA B	ASIA C	ASIA D	Total
C1	2	0	0	0	2
C2	1	0	0	0	1
C3	4	0	1	0	5
C4	2	0	1	0	3
C5	0	0	1	1	2
C6	0	1	0	0	1
C7	0	1	0	0	1
T2	0	0	0	1	1
T3	1	0	0	0	1
T4	0	0	0	1	1
Total	10	2	3	3	18

Data are expressed in absolute numbers.

*ASIA* American Spinal Injury Association.

### Follow-up of TCSI patients

**Figure 3:**
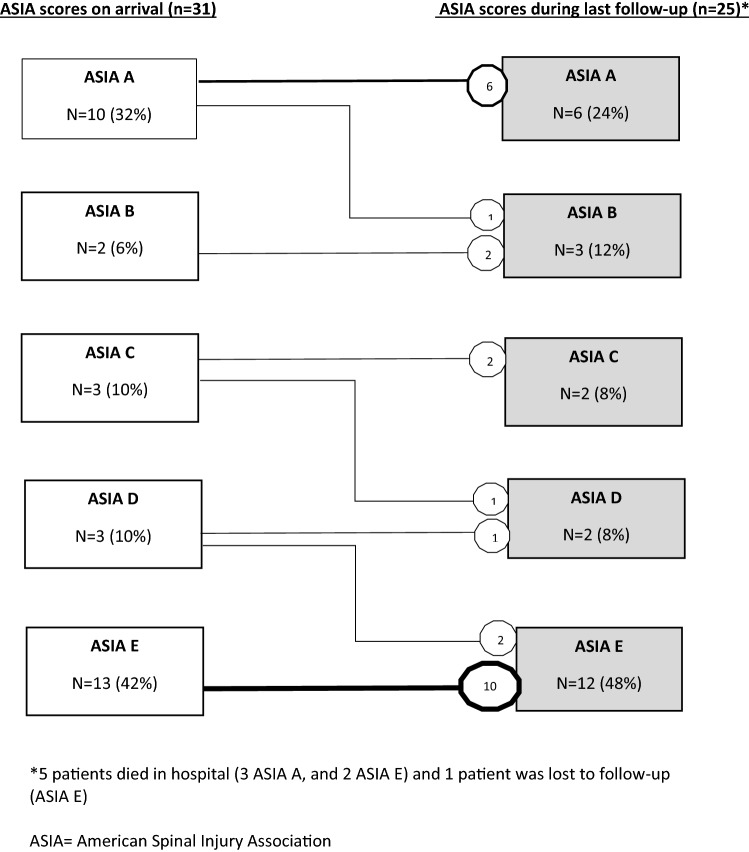
Spinal cord injury severity; relation between ASIA score on arrival and during last follow-up.

Five (16%) TCSI patients died in hospital (3 patients with ASIA A, and 2 patients with ASIA E). Median follow-up time of TCSI patients was 22 (11–29) months. One patient with ASIA E was lost to follow-up. Three patients remained ventilator-dependent at discharge. Figure 3 demonstrates the relation between ASIA on arrival in hospital and ASIA during last follow-up. None of the patients developed deterioration of their spinal cord injury. Four TSCI patients (22%) had amelioration of their spinal cord injury during follow-up; Two patients with TSCI on thoracic spine level improved from ASIA D to normal motor and sensory function (ASIA E), one patient with TSCI on C3 progressed from ASIA A to B, and one patients with TSCI on C3 improved from ASIA C to D. During follow-up period three TCSI patients (10%) died, all with associated spinal cord injury on the level of C3 or C4. One patient died due to sepsis caused by a pressure ulcer, one due to arrhythmias caused by autonomic dysfunction associated with high spinal cord injury, and one died of respiratory insufficiency due to pulmonary complications.

## Discussion

In this study the incidence of severe TCSI in polytrauma was much lower than severe TBI. Cause of death in TCSI was different compared to TBI demonstrating that AIShead based on cervical spine injury is a different entity than AIShead based on TBI.

The incidence of severe TCSI was 6% (31/486) in all polytrauma patients admitted to ICU, and 11% (31/284) in patients with AIShead ≥ 3. Additionally, 9% of patients with severe TCSI had associated severe TBI. The incidence of TCSI and its association with TBI is in accordance with several other reports from literature^1–4,7–10^.

TCSI patients had a lower SBP on arrival in ED. It is likely that the majority of TCSI patients with associated serious spinal cord injury had developed neurogenic shock. However, there was no significant difference in SBP in ED in TCSI patients with and without spinal cord injury. Further, TCSI patients stayed almost twice as long in ICU than TBI patients even though ventilator days and length of hospital stay were comparable. Again, this could not be explained by the presence of spinal cord injury since there was no difference in ventilator days, days in ICU, and days in hospital in TCSI patients with and without spinal cord injury. Possibly, TCSI patients had a higher risk of respiratory problems, needed a tracheostomy more often, and therefore stayed longer in ICU even though they were technically not mechanically ventilated anymore.

In this study detailed data on traumatic cervical spine injuries showed that C2 was the most common location of cervical spine fracture, followed by C6–C7. This is in line with other studies^1,4,21^. More than half the patients suffered from complete spinal cord injury with C3–C4 being the most common location for the spinal cord injury. This complete spinal cord injury rate is higher than reported in previous studies^4,22,23^. This is likely caused by the inclusion criteria of polytrauma patients with associated AIShead ≥ 3. Another explanation could be that there were several TCSI patients with associated high spinal cord injury. In the past these patients would have died prior to arrival to the hospital because of the inability to breath spontaneously. With short transport times and quick access to medical treatment these patients nowadays frequently arrive alive in ED. Consequently, this high cervical spinal cord injury with subsequent inability to breath spontaneously has become one of the most common causes of death in TCSI patients. The high incidence of respiratory insufficiency which was the other major cause of death could be explained by the fact that injuries to the chest were the most frequently and most severely associated injuries in both TCSI and TBI. This association of chest injuries and injuries to the cervical spine and TBI in severely injured have been reported previously as well^22,24^. The differences in cause of death between TCSI and TBI patients were also reflected by the time of death; median time to death was almost twice as long in TCSI patients compared to TBI patients. This was however not statistically significant which was likely caused by the relative low numbers of deceased in TSCI group.These differences in cause and time of death also indicate that AIShead based on TCSI should be treated as a different entity than AIShead based on TBI.

Twenty-two percent of TCSI patients with traumatic spinal cord injury had any improvement in motor and/or sensory function during median follow-up of almost two years; 66% of ASIA D, 9% of ASIA A and 33% of ASIA C patients improved by one grade. This seems lower than reported in the meta-analysis by Khorasanizadeh et al.^25^. This is likely caused by both the inclusion criteria (polytrauma, AIShead ≥ 3, admission to ICU) and the low numbers in this study.

TCSI patients with associated spinal cord injury had similar in-hospital mortality rates than TCSI without spinal cord injury, suggesting that in-hospital care for patients with associated traumatic spinal cord injury was adequate. However, when overall mortality including death during follow-up was calculated, 26% (8/31) of TCSI patients died. Thirty-three percent (6/18) of patients with spinal cord injury died compared to 15% (2/13) without spinal cord injury. This seems to be higher than in polytrauma patients with associated TBI. In a previous study in isolated moderate to severe TBI it was demonstrated that few patients died post hospital discharge^26^.

This high overall death rate shows that patients with associated spinal cord injury have residual problems after discharge from hospital with significant risk at dying from complications related to the immobility caused by the spinal cord injury. This was demonstrated by others as well^27^.

The strength of this study was detailed data of both demographics, physiology, resuscitation and in-hospital outcome in polytrauma patients with AIShead ≥ 3, and an almost 2-year follow-up in severely injured patients with associated traumatic cervical spine injury.

A few limitations need to be acknowledged: Firstly, this was a retrospective analysis of a single center prospective cohort study with relatively low numbers and its accompanying limits. Further, treating clinicians were also the researchers. Additionally, no details on comorbidities were collected nor any post discharge data on polytrauma patients with associated TBI.

To our knowledge, this is the first study in which polytrauma patients with AIShead ≥ 3 based on either TCSI or TBI were compared. Data demonstrated that TCSI patients are a different entity than TBI patients which is clearly shown by differences in cause of death. For future studies it is important to keep in mind that traumatic cervical spinal (cord) injuries have a potentially different threat of life associated with the injury than TBI. To acknowledge these differences and to avoid data misinterpretation injuries to the cervical spine should be distinguished from TBI in morbidity and mortality analysis even though they have similar injury severity according to Abbreviated Injury Scale.

## Data Availability

The dataset supporting the conclusions of this article are available upon reasonable request from the corresponding author.
